# The Post-translational Modifications of Smurf2 in TGF-β Signaling

**DOI:** 10.3389/fmolb.2020.00128

**Published:** 2020-07-07

**Authors:** Yangjinming Bai, Ying Ying

**Affiliations:** ^1^Jiangxi Province Key Laboratory of Tumor Pathogens and Molecular Pathology and Department of Pathophysiology, Schools of Basic Medical Sciences, Nanchang University, Nanchang, China; ^2^Nanchang Joint Program, Queen Mary School, Nanchang University, Nanchang, China

**Keywords:** post-translational modifications, Smurf2, SUMOylation, ubiquitylation, neddylation, phosphorylation, methylation

## Abstract

Smad ubiquitin regulatory factor 2 (Smurf2), an essential negative regulator of TGF-β signaling, ubiquitinates TGF-β receptors (TβRs) and Smad proteins, inducing their proteasomal degradation. Smurf2 plays crucial roles in regulating TGF-β signaling and maintaining normal cellular functions and tissue homeostasis; dysfunction of Smurf2 triggers abnormal TGF-β signaling in pathological states. Smurf2 has been reported as a potentially strong candidate for targeting therapies for related diseases. Recent work has begun to focus on the regulation of Smurf2 itself, and emerging evidence indicates that Smurf2 is regulated by post-translational modifications (PTMs) mechanisms. These mechanisms predominantly regulate the expression level and E3 ligase activity of Smurf2, strongly suggesting that this protein contributes to complicated roles under multiple pathophysiological conditions. In this review, we cover some significant and novel mechanisms of the PTMs that potentially control Smurf2 participation in TGF-β signaling, including ubiquitylation, SUMOylation, neddylation, phosphorylation, and methylation in order to provide a broad view of the depth and sophistication of Smurf2 function in TGF-β regulation, as well as perspectives for future therapeutic directions for its associated diseases.

## Introduction

Signaling mediated by the transforming growth factor β (TGF-β) family controls many cellular responses and diverse biological processes, such as cell growth, differentiation, adhesion, migration, and apoptosis (Drabsch and ten Dijke, 2012). The TGF-β signaling transduction network entails a complex series of protein interactions, including the activation of serine/threonine kinase receptors, SMAD protein phosphorylation and mobilization to the nucleus, and subsequent regulation of transcription factors that modulate target gene expression. Furthermore, dysregulation of TGF-β signaling can lead to various pathologies such as fibrosis, cardiovascular pathology, and cancer (Eichhorn et al., 2012; Iyengar et al., 2015; Shu et al., 2016; He et al., 2017; Chanda et al., 2018). Smad ubiquitin regulatory factor 2 (Smurf2), a HECT (homologous to the E6-accessory protein C-terminus)-type E3 ubiquitin ligase located mainly in the nucleus, has been demonstrated to play pivotal roles in the negative regulation of TGF-β signaling (David et al., 2013). Typically, Smurf2 translocates out of the nucleus in response to the activation of TGF-β receptor and forms a complex with I-Smads to ubiquitinate TGF-β type I receptors (TβRI) and R-Smads, thereby leading to their proteasomal degradation, and subsequently attenuating the TGF-β signaling.

Other biological functions of Smurf2 have been reported in addition to the regulation of TGF-β signaling. Emerging evidence has demonstrated that Smurf2 contributes to genomic stability, cell polarity, tissue homeostasis, and tumorigenesis (Koganti et al., 2018). Smurf2 acts as both a tumor promoter and suppressor. Knock out of Smurf2 results in tumorigenesis in mice (Ramkumar et al., 2012). In contrast, functional Smurf2 inhibits cancer cell proliferation and tumorigenesis via ubiquitination and degradation of several critical cellular proteins, such as Sirtuins (Yu et al., 2020), SIRT1 (Yu et al., 2019), ChREBP (Li et al., 2019), and RNF20 (Manikoth Ayyathan et al., 2020). However, some studies have documented evidence that Smurf2 functions as a tumor promoter rather than a tumor suppressor under some specific circumstances (David et al., 2013). Additionally, high levels of Smurf2 expression have been found in association with several types of cancer (Jin et al., 2009; Klupp et al., 2019) and were correlated with poor prognosis (Fukuchi et al., 2002; Klupp et al., 2019). However, the mechanism underlying these dual roles for Smurf2 in cancer remain poorly understood. Most recently, Emanuelli et al. (2019) found that altered expression and localization may potentially diminish its tumor-suppressive activities. Elucidating the regulatory mechanisms Smurf2 activity and expression is imperative for understanding its role in multiple pathophysiological conditions.

An increasing number of studies have demonstrated that Smurf2 activity and expression are regulated by a series of post-translational modifications (PTMs), including ubiquitylation, phosphorylation, methylation, SUMOylation, and neddylation (Xu et al., 2012). Given that PTMs not only regulate the activity of Smurf2 to control TGF-β signaling but are also involved in the development of several diseases, it is an urgent priority to resolve the underlying mechanisms of how PTMs affect Smurf2 function for identification of potential therapeutic targets. Herein, we review the PTMs that have been thus far reported to affect Smurf2 activity and stability.

## Structure and Functions of Smurf2

**Figure 1:**
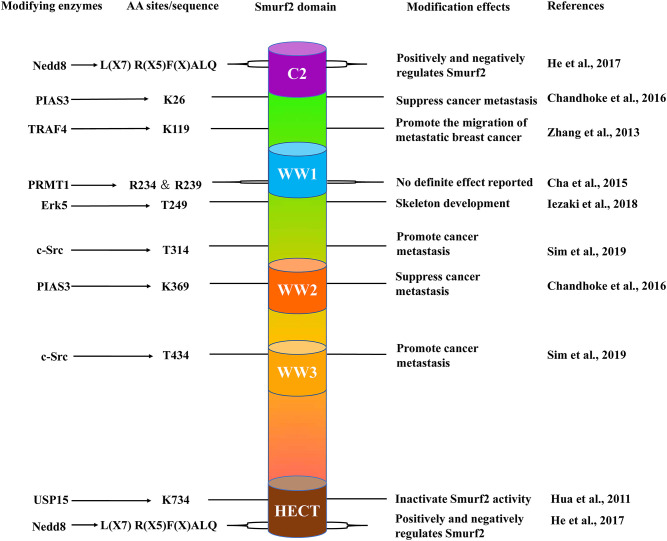
The schematic structure of Smurf2. Smurf2 is composed of an N-terminal C2 domain (purple); three tryptophan-containing WW domains (blue, orange, and yellow) and one C-terminal HECT domain (brown). The locations of specific amino acid sites (center left), the enzymes that target these residues (far left), and the effects of their respective modifications (center right) are included with their corresponding studies (far right).

First discovered in 2000, Smurf2 is a member of the Neural Precursor Cell-expressed Developmentally Down-regulated Protein 4 (NEDD4) subfamily (Kuratomi et al., 2005). The human *smurf2* gene, located on chromosome 17, and Smurf2 protein contain three regions: a C2 domain at the N-terminal, three WW domains containing two conserved tryptophan residues each, and a highly conserved HECT catalytic domain at the C-terminal (Figure 1; Lin et al., 2000). Moreover, the WW domain is responsible for substrate recognition through specific binding to a PPXY motif (Zhu et al., 1999). In the resting state, the C2 domain associates with the HECT domain on Smurf2 to prevent the WW domain from interacting with substrates. This mechanism potentially contributes to maintaining the stable expression of Smurf2 in cells. Furthermore, Smurf2 requires adaptor proteins to facilitate the induction of its active state to proceed with enzymatic interactions with its substrates (Wiesner et al., 2007). To date, many adaptors have been found to interact and promote the function of Smurf2. The first reported, canonical protein adaptor is Smad7. Smad7 binds to Smurf2, forming a complex, to initiate Smurf2 translocation out of the nucleus for targeting of the TGF-β receptor complex for degradation (Kavsak et al., 2000).

As a C2-WW-HECT type E3 ubiquitin ligase, Smurf2 was described as a negative regulator of TGF-β signaling, and a substantial number of reports subsequently demonstrated that Smurf2 primarily targets signaling components and downstream protein expression induced by TGF-β. For instance, Smurf2 not only associates with the I-Smads to down-regulate type I TGF-β receptor (TβRI) and R-Smads (Kavsak et al., 2000; Lin et al., 2000; Zhang et al., 2001) but also degrades SnoN by assembling a complex with Smad2 (Bonni et al., 2001). Moreover, TGF-β was shown to up-regulate the transcription level of Smurf2, thus generating a negative feedback loop for TGF-β signaling (Ohashi et al., 2005). Smurf2 and Smad7 are the strongest negative regulators of TGF-β (Wegner et al., 2012). Notably, the negative feedback loop can be disrupted by Ring finger protein 11 (RNF11) activity, which is overexpressed in cancer cells. RNF11 binds directly to Smurf2, preventing the formation of the Smad7-Smurf2 complex, resulting in constitutive induction of TGF-β signaling (Malonis et al., 2017). This mechanism has major implications for the role Smurf2 in related diseases, such as pancreatic and breast cancer (Seki et al., 1999; Subramaniam et al., 2003).

Previous studies have also demonstrated that Smurf2 is autoinhibited by its C2 domain. The C2 domain interacts with the HECT domain via the catalytic cysteine, thereby inhibiting the formation of the ubiquitin thioester between Smurf2 and its substrates. Notably, Smad7 has been shown to antagonize this process to activate Smurf2 (Wiesner et al., 2007). A mechanistic study also found that, in some circumstances, the Smurf2 WW1 domain associates with the C2-WW1 linker and strongly enhances the C2-HECT interaction, effectively down-regulating its E3 ligase activity. Intriguingly, the WW domain in Smurf1 does not exert this effect. To better understand the role of the WW1 domain in Smurf2, a customized Smurf1 with an additional Smurf2 WW1 domain and a recombinant Smurf2 lacking the WW1 domain were used to determine that Smurf1 carrying a Smurf2 WW1 domain exhibited auto-inhibition, while deletion of the WW1 domain led to Smurf2 activation. The results indicated that the WW1 domain in Smurf2 is essential for its autoinhibition (Ruetalo et al., 2019). In agreement with this finding, in bladder cancer, the C2-HECT interaction between Smad7 and Smurf2 was prevented by an abnormal PTM, a phenomenon which we discuss in further detail below (Sim et al., 2019).

## Ubiquitylation and Deubiquitylation of Smurf2

Ubiquitylation is highly conserved among animal organisms and is fundamental for the regulation of protein stability. As an E3 ligase, Smurf2 can polyubiquitinate TβRI as well as Smad2/3 to attenuate TGF-β signaling. Similarly, Smurf2 is also subjected to negative regulation by ubiquitylation via other E3 ligases, such as tumor necrosis factor receptor-associated factor 4 (TRAF4) (Zhang et al., 2013), Tribbles homolog 3 (TRB3) (Hua et al., 2011) and tetratricopeptide repeat domain 3 (TTC3) (Kim et al., 2019). A recent study revealed that Smurf2 is both the substrate and the direct target of TRAF4 through TRAF4 interactions with the Smurf2 C2 and WW domains. Overexpression of wild-type TRAF4 in HEK-293T cells was found to significantly enhance the polyubiquitylation of Smurf2, while TRAF4 deletion stabilized Smurf2, thus suggesting that TRAF4 contributes to polyubiquitylation and degradation of Smurf2 (Zhang et al., 2013).

Similar to TRAF4, the silencing of TRB3 led to the up-regulation of Smurf2 protein levels, but not its mRNA levels. In contrast, TRB3 overexpression decreased Smurf2 protein levels (Hua et al., 2011). Furthermore, immunoprecipitation assays confirmed that TRB3 promoted Smurf2 ubiquitylation and degradation. In addition, TTC3 was found to interact with the catalytic domain of Smurf2, directly triggering Smurf2 ubiquitylation (Kim et al., 2019). Another report on Smurf2 degradation found that F-box and LRR domain-containing protein 15 (FBXL15) targeted Smurf2, leading to its ubiquitylation and degradation (Cui et al., 2011). Additionally, Smad7 was shown to induce Smurf2 E3 ligase activity as well as mediate Smurf2 autoubiquitylation and degradation via interaction with the HECT domain (Ogunjimi et al., 2005).

Deubiquitinating enzymes function in the reversal of the ubiquitylation process. For example, ubiquitin-specific protease 15 (USP15) can directly deubiquitinate Smurf2, thus causing the loss of Smurf2 catalytic activity (Iyengar et al., 2015). Further studies found that USP15 targets the essential catalytic site residue, Lysine 734, for deubiquitination (Iyengar et al., 2015). Notably, unlike USP15, USP11 appears to indirectly enhance the ubiquitylation of Smurf2, although the mechanism remains unclear (Iyengar et al., 2015).

Accumulating evidence indicates that ubiquitylation and degradation of Smurf2 promote the development of some diseases, such as fibrosis and cancer. A recent study in HepG2 cells showed that TRB3 promoted cancer cell migration and invasion through enhancement of Smurf2 ubiquitylation (Hua et al., 2011). In BEAS-2B cells and NHLFs cells, TTC3 was found to induce Smurf2 proteasomal degradation by ubiquitination in a Lys48-linked manner, hence contributing to TGF-β-induced epithelial-mesenchymal transition (EMT) and myofibroblast differentiation (Kim et al., 2019). Moreover, TRAF4 was found to promote the migration of metastatic breast cancer through Lys48-linked ubiquitylation of Smurf2 at Lys 119 (Zhang et al., 2013). These findings strongly suggest that targeting Smurf2 may be a viable strategy for the treatment of its related diseases.

## SUMOylation of Smurf2

The small ubiquitin-like modifier (SUMO) system is a post-translational modification system associated with ubiquitylation. SUMOylation of Smurf2 was first observed at K26 and K369 by SUMO E2 Ubc9 and E3 enzyme, the protein inhibitors of activated STATs 3 (PIAS3) in NMuMG epithelial cells (Chandhoke et al., 2016). Moreover, the SUMOylation of Smurf2 was found to be reversed by sentrin-specific proteases (SENP) 1 and SENP2 but not SENP3, suggesting that SENP1 and SENP2 might be deSUMOylases for Smurf2 (Chandhoke et al., 2016).

SUMOylation modification has been shown to enhance the Smurf2-mediated induction of TβR degradation (Chandhoke et al., 2016). However, a Smurf2 double mutant carrying arginine replacements of Lysine 26 (K26R) and 369 (K369R) (Smurf2KdR), which was still capable of binding activated TβR similar to wild-type Smurf2, lost its ability to attenuate TGF-β signaling and failed to inhibit EMT. This finding suggested that the SUMOylation of Smurf2 is essential for its suppression of TGF-β-induced EMT.

Notably, Smad7 binds to Smurf2 through association with NTD–HECT, and thus promotes the autoubiquitylation of Smurf2 by recruiting E2s (Wiesner et al., 2007). Since KdR mutation has little effect on the interaction of Smurf2 to Smad7, ostensibly SUMOylation does not affect its autoinhibition (Kavsak et al., 2000; Wiesner et al., 2007; Ruetalo et al., 2019). Further investigation revealed that PIAS3 potentially maintained a non-invasive phenotype through Smurf2 SUMOylation in human MDA-MB-231 breast cancer cells, indicating an anti-metastatic activity of SUMOylated Smurf2 (Chandhoke et al., 2017).

## Phosphorylation of Smurf2

Phosphorylation is a prevalent PTM that regulates protein function. Akt was the first kinase identified to phosphorylate Smurf2, which led to the down-regulation of its protein levels through ubiquitin/proteasome-mediated degradation. However, the use of an anti-phospho-Akt substrate motif (RXX^*^/T^*^) antibody to detect Smurf2 phosphorylation in that study prevented the identification of specific phosphorylation sites (Choi et al., 2014). Recently, extracellular signal-regulated kinase 5 (Erk5) was found to phosphorylate Smurf2 at Thr249, thereby enhancing its ability to target Smad 1, Smad2, and Smad3 for ubiquitylation and proteasome-mediated degradation. Moreover, a Smurf2 T249A mutant, defective for phosphorylation by ERK5, was not able to induce Smad protein ubiquitylation, whereas a T249E mutation, which mimicked phosphorylation by ERK5, caused extensive Smad ubiquitylation, irrespective of the presence or absence of ERK5. These findings implied that, under certain conditions, ERK5-mediated phosphorylation is a prerequisite for ubiquitin E3 ligase activity by Smurf2 (Iezaki et al., 2018).

Work by other groups has shown that c-Src phosphorylated Smurf2 at Tyr314/Tyr434. This activity inhibited Smad7 binding and maintained Smurf2 in a closed, inactive conformation by promoting its own C2-HECT domain interaction. A conversion mutation of Tyr314/Tyr434 to glutamines, which mimicked phosphorylation by c-Src, completely abrogated Smurf2-mediated TβRI degradation. In contrast, a phosphorylation-defective mutant generated by conversion of the Tyr314/Tyr434 residues to phenylalanine nullified the ability of c-Src to downregulate Smurf2 activity (Sim et al., 2019). Taken together, these findings demonstrated that distinct phosphorylation patterns induced by various protein kinases result in different outcomes for the regulation of Smurf2.

## Methylation of Smurf2

Smurf2 was also found to be methylated by protein arginine methyltransferase 1 (PRMT1) (Cha et al., 2015), with methylation sites identified within the amino acid region 224-298, including residues Arg232, Arg234, Arg237, and Arg239. Among these four sites, Arg234 and Arg 239 are specific to PRMT1. Moreover, PRMT1 knockdown led to the up-regulation of Smurf2 protein levels, which implied that methylation of Smurf2 by PRMT1 is involved in the maintenance of Smurf2 stability. However, wild-type PRMT1 overexpression or catalytic inactivation of PRMT1 exerted no detectable effects on the inhibitory role of Smurf2 in TGF-β signaling (Cha et al., 2015).

## Neddylation of Smurf2

Protein neddylation is an essential biological process in which Nedd8 (neural precursor cell expressed developmentally downregulated protein 8), a ubiquitin-like protein, is activated by Nedd8 E1 and E2 enzymes and then conjugated to lysine residues in the target protein by Nedd8 E3 enzyme (Kamitani et al., 1997; Rabut and Peter, 2008; Zhou et al., 2018). In addition to the cullin-RING E3 ligase (CRL) family members, which are the most widely studied substrates known to be activated by neddylation, an increasing number of non-cullin proteins have been reported to be modified by Nedd8 (Xie et al., 2014; Enchev et al., 2015; Shu et al., 2016; He et al., 2017; Zhou et al., 2018).

Both Smurf1 and Smurf2 were found to be modified by the neddylation system (He et al., 2017). For Smurf1, covalent binding to Nedd8 results in a Nedd8-thioester intermediate, which consequently causes the neddylation of multiple lysine residues, notably in the C2 and HECT domains, as well as in the WW-HECT linker. However, Smurf2 is neddylated primarily at sites in the HECT region. Recently, a conserved non-covalent Nedd8 binding sequence, L(X7)R(X5)F(X)ALQ, was verified in the catalytic HECT domain of both Smurf1 and Smurf2. Moreover, the conversion of these conserved residues to alanine in both the N- and C-lobes of the Smurf2 HECT domain prevented its interaction with Nedd8 and attenuated its ability to induce Smad3 degradation. These results suggested that the non-covalent binding with Nedd8 is essential for Smurf2 regulation of BMP/TGF-β signaling. Intriguingly, neddylation promotes Smurf2 degradation while also enhancing its E3 ligase activity (Shu et al., 2016; He et al., 2017).

In addition, Nedd8 overexpression was shown to significantly increase the poly-ubiquitylation of Smurf2, thus enhancing its turnover by proteasomal degradation. In contrast, Smurf2 ubiquitylation was unaffected by overexpression of a Nedd8 ΔGG mutant, which lacked the ability to covalently conjugate Smurf2. Moreover, neddylation also promoted the ubiquitylation of a ligase-inactive mutant of Smurf2, which differed from Smurf1 due to its neddylation-augmented auto-ubiquitylation (Shu et al., 2016). Furthermore, *in vitro* experiments demonstrated that Smurf2 was effectively neddylated in the absence of any other Nedd8 E3 ligase, strongly suggesting that Smurf2 itself is a potential neddylation E3 ligase, and which supports its autoneddylation. However, *in vivo* neddylation assays with HA-Nedd8 and Myc-tagged-Smurf2 mutants carrying alanine conversions of each cysteine residue in the HECT domain, co-transfected into failed to identify the responsible, active site since all mutants were neddylated to the same extent as wild-type Smurf2 (Shu et al., 2016). Given that Smurf1 was demonstrated to function as a Nedd8 ligase and also to interact with Smurf2, it is possible that the above mentioned Myc-tagged-Smurf2 mutants were potentially neddylated by Smurf1, although this hypothesis requires further investigation.

## Conclusion and Discussion

**Figure 2:**
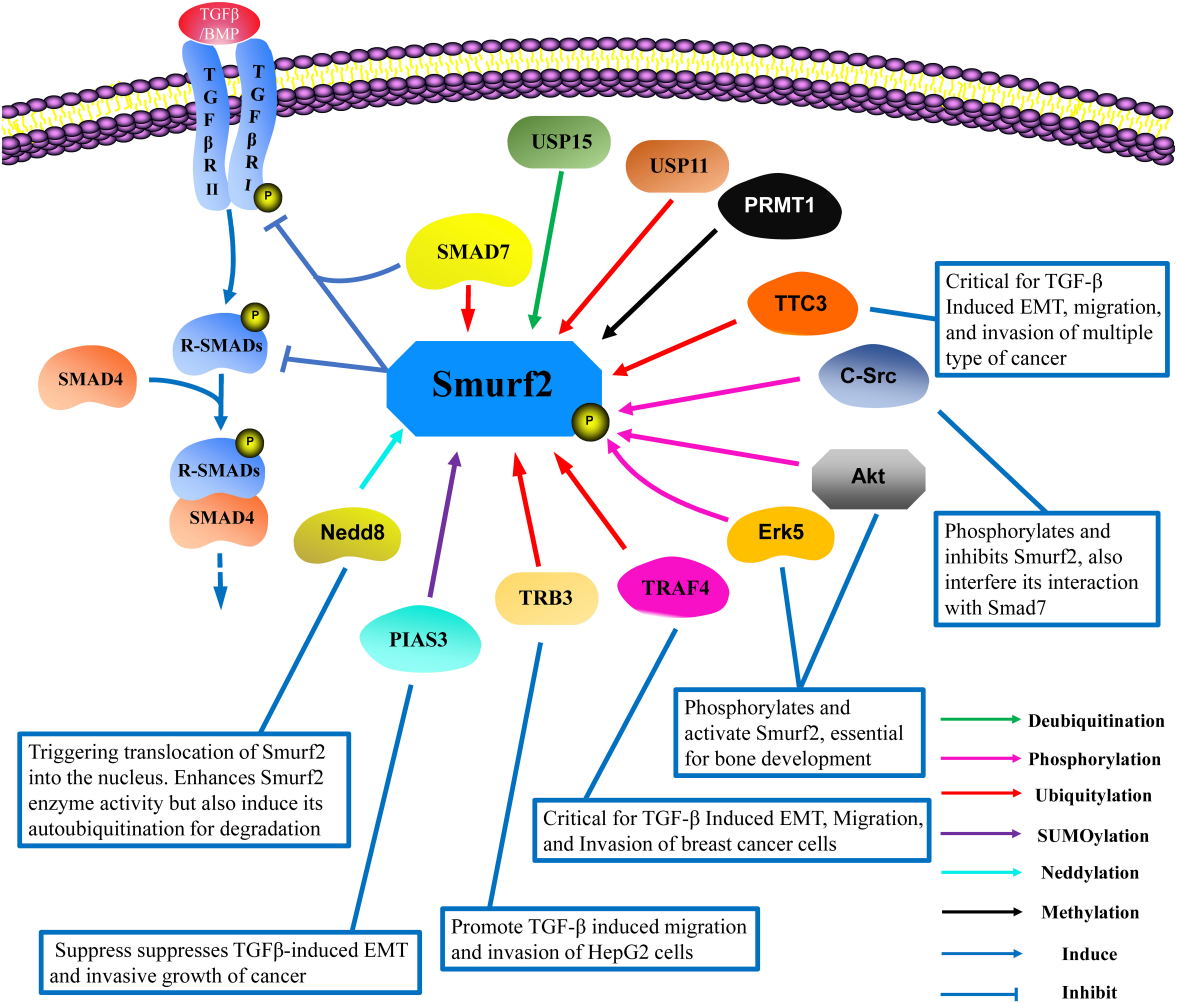
The interacting protein network and post-translational modifications of Smurf2. Smurf2 works in conjunction with SMAD7 to degrade R-Smads and TβRI. To date, the reported post-translational modifications of Smurf2 include ubiquitylation (red lines), deubiquitylation (green lines), SUMOylation (purple lines), neddylation (light blue lines), phosphorylation (pink lines), and methylation (black lines). TRAF4, TRB3, and TTC3 induce Smurf2 degradation in a ubiquitin-dependent manner. USP15 can deubiquitinate Smurf2, while USP11 can increase the ubiquitylation level of Smurf2, although the mechanism remains unknown. Neddylation by Nedd8 enhances Smurf2 function, but also induces Smurf2 degradation. PIAS3 mediates the SUMOylation of Smurf2, which promotes Smurf2 attenuation of TGF-β signaling. Methylation by PRMT1 exerts no clear effect on Smurf2 functions. Phosphorylation by Erk5 and Akt enhance Smurf2-mediated interference with TGF-β signaling, which is essential for bone development. Additionally, phosphorylation by c-Src inhibits the activation of Smurf2 in cancer development, thus inducing epithelial mesenchymal transition (EMT). USP, Ubiquitin-specific protease; TRAF4, Tumor necrosis factor receptor-associated factor 4; TRB3, Tribbles homolog 3; TTC3, Tetratricopeptide repeat domain 3; PIAS3, The protein inhibitors of activated STATs 3; Erk5, Extracellular signal-regulated kinase 5; PRMT1, Protein arginine methyltransferase 1; Nedd8, Neural precursor cell expressed developmentally downregulated protein 8.

Smurf2 is a C2-WW-HECT-domain E3 ubiquitin ligase that contributes pivotal functions in a variety of physiological and pathological processes through the regulation of protein stability and TGF-β pathway signaling (Koganti et al., 2018). Multiple reports have demonstrated that Smurf2 undergoes extensive post-translational modifications that regulate its function and stability, and some of which have identified specific amino acid site targets for modification (Figure 2).

Based on the available evidence, the PTM system governing Smurf2 activity and accumulation involves a complex and sophisticated suite of interacting proteins. Together, several studies have demonstrated that TTC3, TRAF4, and TRB3 promote Smurf2 ubiquitylation and lead to its degradation. All three ubiquitylation enzymes reportedly promote tumor growth, invasiveness, or EMT. In light of results demonstrating that ubiquitylation is a reversible process, deubiquitylation enzymes have been proposed as a means of reducing ubiquitylation levels of Smurf2 for interference with tumor growth. However, it was also reported that USP11 increased the ubiquitination level of Smurf2 through an unknown mechanism, while the high expression of another deubiquitylation enzyme USP15 was correlated with high TGF-β activity (Iyengar et al., 2015).

Modification by PIAS3 results in Smurf2 SUMOylation, thereby enhancing its E3 ligase activity. In addition, Smurf2 was shown to be phosphorylated by Akt, Erk5, and c-Src, although activity by each of these proteins resulted in different regulatory outcomes. Specifically, Akt-mediated phosphorylation induced Smurf2 degradation, and ERK5-mediated phosphorylation increased its E3 ligase activity to degrade Smad proteins, while c-Src-mediated phosphorylation prevented Smurf2 activation by Smad7 and induced its proteasomal degradation. Furthermore, neddylation was revealed to promote both the ubiquitin ligase activity and the degradation of Smurf2, while Smurf2 methylation mediated by PRMT1 apparently exerted little effect on its function. These studies together present an intricate system of Smurf2 regulation by PTMs, and provide a strong basis for in-depth interrogation of the specific mechanisms by which Smurf2 dysregulation can lead to pathogenic outcomes. Given the complexity of PTM-mediated regulation of Smurf2 activity and stability, it is also unsurprising that there are endogenous mechanisms for reversal of these modifications, or that there is potentially substantial overlap or redundancy in protein interactions within this network that can lead to crosstalk or dysregulation.

Furthermore, multiple PTMs can positively or negatively influence each other's activity, i.e., through PTM crosstalk. Under certain conditions, PTM crosstalk may potentially function to maintain cellular proteostasis, that is, the capacity to adapt to stresses or stimuli while protecting the normal function of individual proteins (Frauke and Vertegaal, 2016). In the case of Smurf2, a body of work has shown that both phosphorylation and neddylation promote its ubiquitylation. However, it remains unclear if there are other mechanisms mediated by PTM crosstalk that can contribute to disease development and progression, and if so, by what underlying mechanisms they are controlled. Additionally, future work will also identify whether other modifications, such as acetylation and glycosylation, play a role in modulating Smurf2 activities.

In conclusion, PTMs play central roles in the regulation of the many functions of Smurf2. A thorough and comprehensive understanding of these roles and the mechanisms by which these PTMs control Smurf2 is critical for understanding the biological and pathological networks in which Smurf2 participates. Moreover, these PTMs will likely prove invaluable for the identification of novel therapeutic targets for diseases caused by dysregulation of TGF-β signaling, such as cancer and fibrosis.

## Author Contributions

YB contributed to manuscript preparation and editing. YY contributed to literature research, revise, and final approval of the article. All authors contributed to the article and approved the submitted version.

## Conflict of Interest

The authors declare that the research was conducted in the absence of any commercial or financial relationships that could be construed as a potential conflict of interest.

## Abbreviations

TGF-β: Transforming growth factor β
PTM: Post-translational modification
Smurf2: Smad ubiquitin regulatory factor 2
TβR: TGF-β receptor
HECT: Homologous to the E6-accessory protein C-terminus
RNF: RING finger protein
ChREBP: Carbohydrate response element-binding protein
SIRT1: The NAD-dependent deacetylase sirtuin 1
FBXL15: F-box and LRR domain-containing protein 15
USP: Ubiquitin-specific protease
NEDD4: Neural Precursor Cell-expressed Developmentally Down-regulated Protein 4
RNF11: Ring finger protein 11
TRAF4: Tumor necrosis factor receptor-associated factor 4
TRB3: Tribbles homolog 3
TTC3: Tetratricopeptide repeat domain 3
SUMO: Small ubiquitin-like modifier
PIAS3: The protein inhibitors of activated STATs 3
Erk5: Extracellular signal-regulated kinase 5
HGF: Hepatocyte growth factor
PRMT1: Protein arginine methyltransferase 1
Nedd8: Neural precursor cell expressed developmentally downregulated protein 8
UBL: Ubiquitin-like protein
CRL: Cullin-RING E3 ligase.
